# Detection of Colistin Heteroresistance in Carbapenem-Resistant *Pseudomonas aeruginosa* Clinical Isolates in Iran

**DOI:** 10.1155/ijm/5571153

**Published:** 2025-10-06

**Authors:** Zahra Riahi Rad, Zohreh Riahi Rad, Hossein Goudarzi, Mehdi Goudarzi, Parisa Pourdehghan, Masoud Kargar, Saeed Shams, Ali Hashemi

**Affiliations:** ^1^Department of Microbiology, School of Medicine, Shahid Beheshti University of Medical Sciences, Tehran, Iran; ^2^Department of Parasitology, Iranian Research Center of Ticks and Tick-Borne Diseases, Faculty of Veterinary Medicine, University of Tehran, Tehran, Iran; ^3^Thalassemia and Hemoglobinopathy Research Center, Health Research Institute, Ahvaz Jundishapur University of Medical Sciences, Ahvaz, Iran; ^4^Cellular and Molecular Research Center, Qom University of Medical Sciences, Qom, Iran

**Keywords:** antibiotic resistance, carbapenem-resistant *P. aeruginosa* (CRPA), colistin, heteroresistance, population analysis profiles, *Pseudomonas* aeruginosa

## Abstract

**Background:**

Since antimicrobial resistance is a rising danger and a serious threat to the health of humans, recent studies have revealed that routine tests, such as minimum inhibitory concentration (MIC), are not enough to detect heteroresistance (HR) phenotype (i.e., phenotypic heterogeneity in antibiotic susceptibility in the identical bacterial population), and medical laboratories require awareness raising to face it. Colistin is regarded as the final therapeutic option for infections caused by carbapenem-resistant gram-negative bacteria. In this study, we explored the presence of HR to colistin in carbapenem-resistant *Pseudomonas aeruginosa* isolates in Iran.

**Methods:**

From 2019 to 2020, 100 *P. aeruginosa* clinical isolates were gathered from hospitals in various regions in Iran. In the present study, antibiotic susceptibility testing was used to determine antibiotic resistance. The population analysis profile (PAP) test was conducted to measure HR. Additionally, PCR was carried out to detect metallo-*β*-lactamase genes, including *bla*_NDM-1_ and *bla*_IMP_, and *bla*_VIM_ genes of colistin heteroresistant isolates.

**Results:**

As a result, of the 100 *P. aeruginosa* isolates that were tested by AST, 66 were resistant to carbapenem antibiotics. Here, we found that 62 carbapenem-resistant *Pseudomonas aeruginosa* (CRPA) isolates were susceptible to colistin, while four isolates were resistant to colistin. Regarding the PAP assay, we identified eight heteroresistant isolates, of which three and two isolates carried *bla*_NDM-1_ and *bla*_IMP_ genes, respectively. Furthermore, the HR isolates demonstrated a stable phenotype after seven subcultures in Mueller Hinton agar (MHA) without antibiotics.

**Conclusions:**

Finally, our study highlights the interplay between HR and its diagnosis methods. Since it is difficult to detect heteroresistant isolates by routine tests in clinical laboratories, it might be misclassified as susceptible and lead to challenges for clinicians and their patients.

## 1. Introduction

Antimicrobial resistance (AMR) is one of the most serious global health threats related to an estimated 4.95 million deaths in 2019 [1]. The comprehensive worldwide study conducted by Murray et al. about AMR demonstrate that infections due to antibiotic-resistant pathogens are one of the main causes of death for people of all ages [1]. A study conducted in 2016 estimated that by 2050, 10 million people could die as a result of AMR every year [2].

*Pseudomonas aeruginosa* is a gram-negative, nonfermenting, opportunistic bacterium and the most common multidrug-resistant (MDR) pathogen in hospitalized patients, which prolongs hospitalization and is associated with severe morbidity and mortality [3]. Based on the 2024 World Health Organization report on bacterial priority pathogens list, *P. aeruginosa* was placed in the high group [4]. Carbapenems (a class of *β*-lactam antibiotics) are extensively utilized to treat severe infections, including patients with infections caused by multidrug-resistant *P. aeruginosa* (MDRPA). Ultimately, this class's widespread and irresponsible use has increased carbapenem resistance worldwide. One of the mechanisms of resistance to carbapenems in *P. aeruginosa* is the acquisition of transferable carbapenemase-encoding genes, including the metallo-*β*-lactamases (MBLs) [5].

Likewise, there has been a revival of interest in using old antibiotics, for example, polymyxins (e.g., polymyxin B and colistin [CO]) [6]. CO resistance in *P. aeruginosa* is related to the addition of 4-amino-L-arabinose (L-Ara4N) or phosphoethanolamine (pEtN) enzymes to the lipid A moiety of lipopolysaccharide (LPS), which leads to a decrease in the net negative charge of the outer membrane. The regulatory two-component systems (TCSs) involved in lipid A modification and subsequent CO resistance in *P. aeruginosa* include PhoP-PhoQ (PhoPQ) and PmrA-PmrB (PmrAB). Recent evidence shows that other ParRS and CprRS TCSs (which function to sense ions and cationic antimicrobial peptides, regulating the expression of the *arnBCADTEF* operon, which encodes enzymes responsible for modifying LPS) are involved in CO resistance in *P. aeruginosa* [7].

Identical bacterial cells within a population can demonstrate phenotypic heterogeneity in antibiotic susceptibility, which causes challenges in definitively categorizing bacteria as either susceptible or resistant [8]. Bacteria can acquire resistance genes through horizontal gene transfer or mutation in native genes, which is deemed a stable characteristic. However, this trait can be unstable and appear only in small fractions of a population, leading to phenotypic heterogeneity in the bacterial population [9].

There is increasing concern about the development of distinct resistance mechanisms in clinical isolates, with antibiotic heteroresistance being an example of this phenomenon. The most comprehensive description of heteroresistance is the existence of a heterogeneous bacterial population with one or more subpopulations representing higher levels of antibiotic resistance than the parental population [8]. This phenotype has been identified in most antibiotic classes, including carbapenems and polymyxins (CO and polymyxin B). Several studies have investigated polymyxin heteroresistance in gram-negative species, including *Acinetobacter baumannii*, *Klebsiella* spp., *Enterobacter* spp., *P. aeruginosa*, and *Salmonella enterica* subsp. *enterica* serovar Typhimurium [8].

Despite CO's efficacy against *P. aeruginosa*, CO heteroresistance needs more attention. Due to its characteristic of phenotypic and genetic instability of heteroresistance, it is challenging to diagnose and investigate it [10]. While the description of this heteroresistance phenotype in *P. aeruginosa* is rarely reported, this phenomenon has been investigated in a limited number of studies because it cannot be accurately evaluated using common clinical diagnostic tests such as the antibiogram, minimum inhibitory concentration (MIC), and *E*-test [11]. The gold standard method for detecting resistant subpopulations is the population analysis profile (PAP) test, as it allows for the detection and quantification of resistant subpopulations [8].

We need more comprehensive studies to further understand the impact of heteroresistance on treatment, as it has created diagnostic and therapeutic dilemmas for clinicians. Since evidence shows that heteroresistance can play a role in unexplained treatment failures, diagnosing this phenotype is an essential prerequisite for choosing the appropriate antibiotic to achieve successful treatment outcomes [8]. This mechanism in bacteria is different, consisting of the activation of TCSs PmrAB and PhoPQ in *P. aeruginosa*, *Enterobacter cloacae*, *Klebsiella pneumoniae*, and *A. baumannii*; biofilm formation and capsule hyperproduction in *K. pneumoniae*; and soxRS-regulated (superoxide response regulon positively controls) overexpression of the acrAB-tolC efflux pump in *Enterobacter asburiae* and *E. cloacae* [6]. However, reports of heteroresistance to CO in *P. aeruginosa* are sporadic, and its mechanism has not been extensively investigated. In this study, we investigated CO heteroresistance in carbapenem-resistant *P. aeruginosa* (CRPA) in Iran and emphasized the importance of identifying this phenomenon.

## 2. Materials and Methods

### 2.1. Clinical Data and Bacterial Strains

During this study, we tested 100 nonduplicated *P. aeruginosa* isolates collected between 2019 and 2020 from various clinical samples of hospitalized patients in 10 cities (Tehran, Birjand, Ahvaz, Ilam, Babol, Bandar Abbas, Hamedan, Gorgan, Qom, and Tabriz) in Iran. The identities of the *P. aeruginosa* isolates were confirmed using the following standard biochemical tests: growth on Simmon citrate medium, growth on Cetrimide agar, cytochrome oxidase activity, pigment production, reaction on triple sugar iron (TSI) agar, motility on SIM medium, and indole production (Merck, Darmstadt, Germany) [12]. All isolates were maintained at −70°C in trypticase soy broth (Merck, Germany) containing 20% glycerol before the study.

### 2.2. Antimicrobial Susceptibility Testing (AST)

We performed AST using the Kirby–Bauer disk diffusion technique on MHA (Merck, Germany) plates, and the results were interpreted according to the recommendations of the Clinical and Laboratory Standards Institute (CLSI) 2023 guidelines [13]. The antibiotic disks (Mast, Company) employed in this test were as follows: piperacillin (PRL, 100 *μ*g), aztreonam (ATM, 10 *μ*g), piperacillin–tazobactam (PTZ, 110 *μ*g), ceftazidime (CAZ, 30 *μ*g), cefepime (CPM, 30 *μ*g), imipenem (IMI, 10 *μ*g), meropenem (MEM, 10 *μ*g), doripenem (DOR, 10 *μ*g), ciprofloxacin (CIP, 5 *μ*g), levofloxacin (LEV, 5 *μ*g), amikacin (AK, 30 *μ*g), and tobramycin (TN, 10 *μ*g). In vitro, the MICs were determined for CAZ, CIP, MEM, IMI, and CO (Sigma-Aldrich) against *P. aeruginosa* using the broth microdilution test according to the CLSI guidelines (2023) [13]. All CO-susceptible isolates (MIC ≤ 2 mg/L) underwent testing for heteroresistance [14]. Quality control of AST was conducted using *P. aeruginosa* ATCC 27853.

### 2.3. Phenotypic Detection of Carbapenemase

The detection of the carbapenemase in CO-heteroresistant *P. aeruginosa* was based on the mCIM test following the 2023 CLSI guidelines [13]. To determine MBL, a combined disk diffusion test (CDDT) was conducted [15]. For this purpose, *P. aeruginosa* PA40 (Accession Number: KM359725) and *P. aeruginosa* ATCC 27853 were used as positive and negative control strains for MBL production, respectively [16].

### 2.4. PAPs

The PAP method is the gold standard for investigating heteroresistance to CO in CRPA isolates [8]. To ensure the accuracy of the experiment, the PAP test was repeated three times. The PAP method, using100 *μ*L of serial dilutions of the bacterial cell suspension on MHA plates, both with and without CO at the following increasing concentrations: 0.5, 1, 2, 4, 8, 16, and 32 mg/L was performed. After incubating the plates for 48 h at 37°C, colony-forming units (CFUs) were enumerated [6]. According to Andersson et al., CO heteroresistance can be defined as the presence of CO-susceptible isolates and the growth of a CO-resistant subpopulation of cells at a CO concentration at least eightfold above the MIC of the parental population, at a frequency of ≥1 × 10^−7^ [8]. The frequency of the CO-resistant subpopulation was determined using the following calculation [17]:

 $$\begin{array}{c} \frac{\left(\text{Number of colonies on colistin plate}\times \text{dilution factor}\right)}{\left(\text{Number of colonies on antibiotic free plate}\times \text{dilution factor}\right)}. \end{array}$$

Subsequently, the stability of the resistant subpopulations by selecting a single colony from the plate with the highest antibiotic concentration was measured for seven consecutive generations on antibiotic-free MHA. Then, the MIC of CO was reevaluated.

### 2.5. PCR Amplification and Sequencing Technique

The study used the manufacturer's protocol from the DNA extraction kit to extract heteroresistant isolates DNA (GeNet Bio Company, Daejeon, Korea; Cat. No, K-3000). PCR was performed to detect MBL genes, including *bla*_NDM-1_ and *bla*_IMP_, and *bla*_VIM_ genes of CO-heteroresistant isolates [16].

PCR reaction was conducted in a total volume of 25 *μ*L containing 3 *μ*L (20 ng) of DNA template and 12.5 *μ*L of 2× Master Mix (SinaClon-Iran, CAT. No., PR901638), including 3 mmol/L MgCl_2_, 0.4 mmol/L dNTPs, 1× PCR buffer, 0.08 IU Taq DNA polymerase, 1 *μ*L of 10 pmol of each primer, and 7.5 *μ*L of sterile distilled water. The reaction was performed on a thermal cycler (Bio Intellectica) using the following cycling conditions: an initial denaturation for 5 min at 94°C, followed by 36 cycles of denaturation 45 s at 94°C, 45 s for annealing at different temperatures, and extension at 72°C for 45 s, with a final extension of 5 min at 72°C. Products from PCR reactions were electrophoresed on a 1%–1.5% agarose gel, visualized by DNA Safe stain (SinaClon), and photographed under UV light (Gel Documentation System). DNA ladder (100 bp Plus) was used as a size marker (SinaClon).

PCR products were purified using the Bioneer Co., Korea Kit. Subsequent DNA sequencing was conducted by the Microgen Company (Korea). Chromas Version 1.45 software was employed to analyze the nucleotide sequences using NCBI BLAST.

## 3. Results

### 3.1. Clinical Data and Bacterial Strains

**Figure 1:**
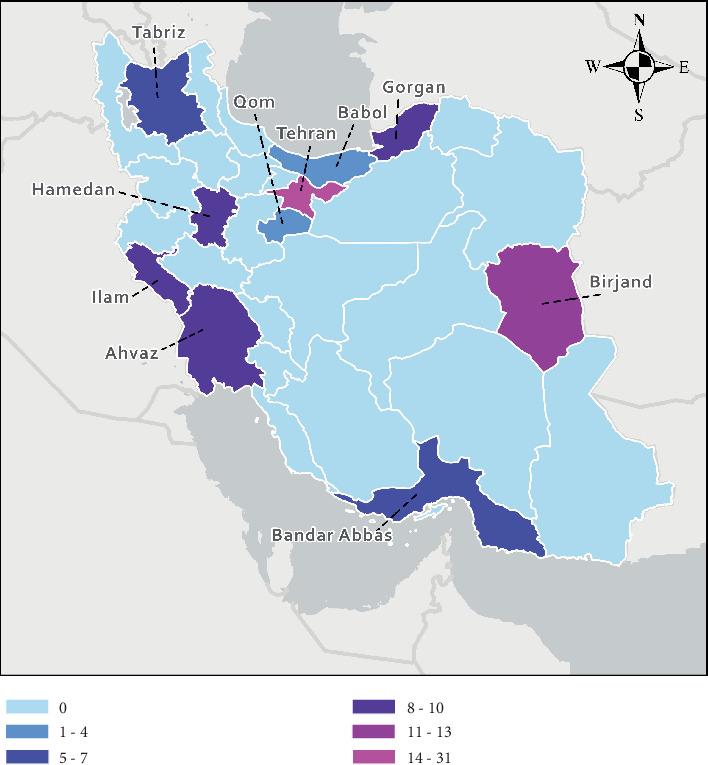
100 *P. aeruginosa* isolates collected from Iran. Thirty-one isolates of Tehran: capital of Iran. Thirty-one isolates of Birjand. Ten isolates of Ilam. Ten isolates of Hamedan. Ten isolates of Gorgan. Nine isolate of Ahwaz. Seven isolates of Bandar Abbas. Five isolates of Tabriz. Four isolates of Babol. One isolate of Qom. The identified heteroresistance samples were isolated from the cities of Tehran, Bandar Abbas, Hamedan, and Gorgan.

A total of 100 *P. aeruginosa* clinical isolates were gathered from 10 cities in Iran within a period of 1 year (2019–2020) (Figure 1). Of the 100 isolates obtained, 49% (*n* = 49) were female and 51% (*n* = 51) male, including 24% (*n* = 24) children. Most isolates were identified from the intensive care units (ICU) 45% (*n* = 45) and burn wards 23% (*n* = 23). The cultural source of the isolates was as follows: urine (*n* = 35), blood (*n* = 30), wound (*n* = 10), bronchoalveolar lavage (*n* = 8), sputum (*n* = 7), throat culture (*n* = 3), trachea (*n* = 3), drainage fluid (*n* = 2), abscess (*n* = 1), and eye infection (*n* = 1).  Table 1 illustrates the demographic data and clinical characteristics of the samples.

### 3.2. Antibiotic Susceptibility and Characteristics of Heteroresistance Among *P. aeruginosa* Isolates

The results obtained from AST showed 66 clinical isolates were resistant to carbapenems (the isolates were resistant to two or three carbapenem antibiotics). As shown in Table 2, there is a significant resistance rate found against MEM 100% (*n* = 66), DOR 97% (*n* = 64), IMI 93.9% (*n* = 62), and PRL 84.8% (*n* = 56). In addition, the data indicate that AK 40.9% (*n* = 27), CAZ 31.8% (*n* = 21), and PTZ 24.2% (*n* = 16) resulted in the highest susceptibility. Based on a definition provided by Magiorakos et al., the total number of CRPA, 19 were MDR. Notably, 41 extensively drug-resistant (XDR) and one pandrug-resistant (PDR) were identified [18]. The MIC test results revealed that a considerable number of CRPA isolates were susceptible to CO 62 (94%) (37 isolates with MIC 0.5 mg/L, 23 isolates with MIC 1 mg/L, and two isolates with MIC 2 mg/L), while four (6%) isolates were resistant (MIC ≥ 4 mg/L) (Table 2). It is worth noting that the MIC values of 66 CRPA isolates are mentioned in Table S1.

**Figure 2:**
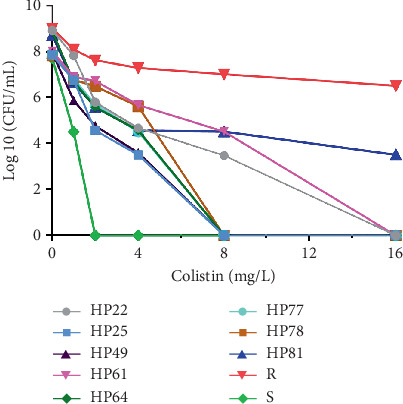
Population analysis profiles (PAP) of eight colistin-heteroresistant *P. aeruginosa*. S, susceptible; R, resistant.

**Figure 3:**
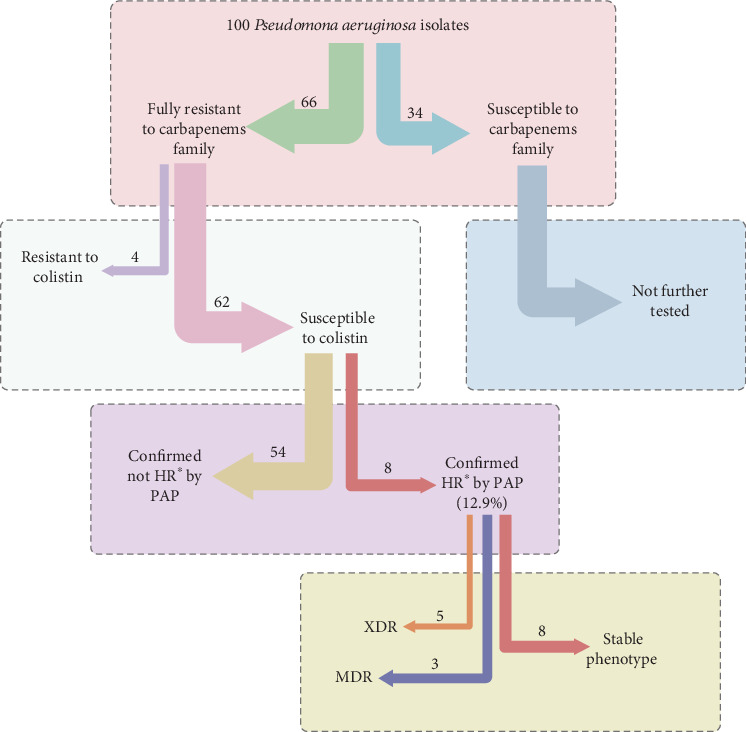
An overview of testes performed and results. ⁣^∗^HR, heteroresistance.

As mentioned earlier, the PAP method is the most reliable test that detects heteroresistance. The PAP results confirmed that eight (12.9%) isolates of 62 CO-susceptible CRPA isolates exhibited a heteroresistant phenotype (Figure 2). The results demonstrated that the growth of the resistant subpopulation had an 8–16-fold increase in CO MICs compared with their respective parent strain. The frequency of resistant subpopulation was 3 × 10^−6^ to 6.1 × 10^−3^. After serial subculture (seven daily) in a CO-free medium, the CO MIC of the resistant subpopulation remained the same, which indicates the stability of the heteroresistance phenotype (Table 3). Data from Table 3 showed, based on the results of PCR analysis, that a few of the resistant subpopulation isolates harbored *bla*_NDM-1_ and *bla*_IMP_ genes. Notably, resistant subpopulation isolates did not carry *bla*_VIM_. AST showed that the CO-heteroresistant *P. aeruginosa* isolates were fully resistance to antibiotics, including MEM, DOR, LEV, and CIP. However, these isolates were susceptible to CAZ and PTZ (Table 4 and Figure 3.

## 4. Discussion

The emergence of antibiotic resistance, an evolving threat, raises questions about effectively treating bacterial infections, and dealing with it involves global efforts [1]. *P. aeruginosa* has a crucial role in causing severe, life-threatening infections in patients, and it is estimated that antibiotic-resistant *P. aeruginosa* could lead to over 300,000 deaths annually [19]. Carbapenems have been considered an option for treating MDRPA infections, but resistance to carbapenem agents is increasing worldwide [20]. Therefore, using polymyxins (e.g., polymyxin B and CO) as a last-line therapy option for CRPA has been proposed [21].

Despite its efficacy, there is an increasing concern about the emergence of the CO-heteroresistant phenomenon. Kayser et al. formally reported the term “heteroresistance” as a concept in 1970 [22]. This phenomenon has been identified in both gram-positive and gram-negative bacteria, wherein the population has a wide variation of resistance to antibiotics [8, 23].

Recent research findings have often reported this phenotype in *P. aeruginosa* to polymyxins [24]. In the present study, 62 of 66 CRPA isolates evaluated were susceptible to CO, and eight isolates showed heteroresistance to CO. To the best of our knowledge, our study is the first evaluation of CO heteroresistance in CRPA in Iran. More recently, Gallardo et al. has reported that the prevalence of heteroresistance is underreported, and it is underappreciated [25].

Estimates of the prevalence of heteroresistance to CO in *P. aeruginosa* have been reported in wide ranges (Table 5). The results of this study indicated a prevalence of CO heteroresistance of 12.9%. Previously, Lin et al. assessed CO heteroresistance in 231 carbapenem-nonsusceptible *P. aeruginosa* isolates from China and reported a prevalence of 4%, which is lower than the 27% and 26% reported by Juhász et al. and Howard-Anderson et al., respectively. [6, 14, 28]. In addition, a study in Brazil reported a polymyxin B heteroresistance prevalence of 4.2% [26]. These rather contradictory results may be due to the differences in detection methods, culture source, geographical location, antibiotic susceptibility pattern, and so on [14].

Our results of the frequency of CO heteroresistance in *P. aeruginosa* were concordant with the definition of Andersson et al. (≥1 × 10^−7^), which was between 3 × 10^−6^ and 6.1 × 10^−3^ [8]. Similarly, Lin et al. reported a CO-heteroresistant proportion in *P. aeruginosa* from 3.61 × 10^−8^ to 7.06 × 10^−6^ [6].

Because antibiotic heteroresistance cannot be explored by routine tests such as *E*-tests and broth microdilution due to the low proportion of heteroresistant cells in standard clinical laboratories [8, 27, 29], its study is limited. However, it has effects on clinical outcomes [30]. This makes the widespread of this phenomenon hidden, and the true prevalence of heteroresistance in infections is underestimated. Despite the complexity and laboriousness of the PAP test, it is the gold standard for detecting heteroresistance [30]. Our results showed no differences from studies previously reported, since the PAP revealed the isolates that were classified as susceptible by broth microdilution were actually heteroresistant [6, 14, 31]. Ozturk and Weiss's results indicated that inconsistency in AST results could be attributed to heteroresistance [32].

Our findings indicate that the heteroresistance phenotype is stable since we detected an increase in CO-MIC values after seven generations on an antibiotic-free medium pressure, which is consistent with the studies of Lin et al. and Hermes et al. [6, 26]. Similar studies have already investigated isolates tested against carbapenems [33].

The main concern about this phenomenon is that it might profoundly impact the efficacy of the treatment, and several studies have given credence to this unfortunate outcome [30, 34–36]. Band et al. pointed out that mice infected with CO-heteroresistant strains failed CO treatment and could not be rescued [35]. Of note, heteroresistance is different from persistence, in which persister cells exhibit very slow growth in the presence of antibiotics [37], while heteroresistant isolates can rapidly enrich in the presence of antibiotics. Persistence by resuming growth after treatment can cause a recurrence of the infection [30].

## 5. Conclusions

This study reveals that the prevalence of CO-heteroresistant isolates among CRPA in different healthcare centers is increasing in Iran. Our study also illustrated that the CRPA has increased. The results of this investigation show how this phenotype is a challenge in clinical microbiology laboratories since it is currently difficult to diagnose heteroresistance with standard tests. Therefore, improving and developing methods that are sensitive enough and have high reproducibility to detect heteroresistance is urgent. Overall, to better understand the epidemiology and the mechanisms of CO heteroresistance and its effects on clinical practice, more studies are needed since the increase in resistance to CO leads to the limitation of treatment choices to control highly resistant infections.

## Figures and Tables

**Table 1:** Demographic data and clinical characteristics of the samples (*n* = 100).

	**Total (** **n** **)**	**%**
Sex		
Male	51	51%
Female	49	49%
City		
Tehran	31	31%
Birjand	13	13%
Ilam	10	10%
Hamedan	10	10%
Gorgan	10	10%
Ahwaz	9	9%
Bandar Abbas	7	7%
Tabriz	5	5%
Babol	4	4%
Qom	1	1%
Culture source		
Urine	35	35%
Blood	30	30%
Wound	10	10%
Bronchoalveolar lavage	8	8%
Sputum	7	7%
Trachea	3	3%
Throat culture	3	3%
Drainage fluid	2	2%
Eye infection	1	1%
Abscess	1	1%
Wards		
ICU	45	45%
Burn	23	23%
Surgery	15	15%
Oncology	8	8%
Internal	7	7%
Cardiology	2	2%

**Table 2:** Antibiotic resistance patterns of CRPA (*n* = 66).

**Antibiotics**	**Disk diffusion ** **n** ** (%)**			**MIC ** **n** ** (%)**		
	**S**	**I**	**R**	**S**	**I**	**R**
Penicillins						
Piperacillin	2 (3)	8 (12.1)	56 (84.8)	—	—	—
Monobactams						
Aztreonam	13 (19.7)	15 (22.7)	38 (57.6)	—	—	—
*β*-Lactam combination						
Piperacillin–tazobactam	16 (24.2)	3 (4.5)	47 (71.2)	—	—	—
Cephems						
Ceftazidime	21 (31.8)	2 (3)	43 (65.2)	23 (34.8)	—	43 (65.2)
Cefepime	6 (9.1)	5 (7.6)	55 (83.3)	—	—	—
Carbapenems						
Meropenem	—	—	100 (66)	—	6 (9.1)	60 (90.9)
Doripenem	1 (1.5)	1 (1.5)	64 (97)	—	—	—
Imipenem	3 (4.5)	1 (1.5)	62 (93.9)	3 (4.5)	8 (12.1)	55 (88.3)
Fluoroquinolones						
Ciprofloxacin	8 (12.1)	7 (10.6)	51 (77.3)	16 (24.2)	—	50 (75.8)
Levofloxacin	10 (15.2)	7 (10.6)	49 (74.2)	—	—	—
Aminoglycosides						
Amikacin	27 (40.9)	2 (3)	37 (56.1)	—	—	—
Tobramycin	13 (19.7)	8 (12.1)	45 (68.2)	—	—	—
Lipopeptides						
Colistin	—	—	—	62 (94)	—	4 (6)

Abbreviations: I, intermediate; R, resistant; S, susceptible.

**Table 3:** Characteristics of the heteroresistant isolates (*n* = 8).

**Strain**	**Gender**	**City**	**Ward**	**Culture source**	**Broth MIC (mg/L)**	**Highest concentration of growth in PAPs (mg/L)**	**Frequency**	**MIC for resistant colonies before daily passages**	**MIC for resistant colonies after 7 days passages**	**Carbapenemase genes**	**Stability**
HP22	Male	Bandar Abbas	ICU	Urine	1	8	3 × 10^−6^	16	16	*bla *_NDM-1_	Stable
HP25	Male	Bandar Abbas	Internal	Urine	0.5	4	4.7 × 10^−5^	4	4	ND	Stable
HP49	Female	Hamedan	ICU	Blood	0.5	4	4.5 × 10^−5^	8	16	*bla *_NDM-1_	Stable
HP61	Male/child	Tehran	ICU	Sputum	1	8	3.3 × 10^−4^	16	8	*bla *_NDM-1_	Stable
HP64	Male	Gorgan	Burn	Urine	0.5	4	5.2 × 10^−5^	8	8	ND	Stable
HP77	Male	Tehran	ICU	Urine	0.5	4	4 × 10^−4^	8	8	*bla *_IMP_	Stable
HP78	Female/child	Tehran	Oncology	Blood	0.5	4	6.1 × 10^−3^	16	16	ND	Stable
HP81	Male	Tehran	Oncology	Urine	1	16	6.4 × 10^−6^	64	64	*bla *_IMP_	Stable

Abbreviation: ND, not detected.

**Table 4:** Antibiotic resistance patterns of the heteroresistant isolates (*n* = 8).

**Strains**	**Disk diffusion**												**mCIM**	**MBL**	**Resistance type**
	**TN**	**CIP**	**IMI**	**MEM**	**DOR**	**AK**	**PRL**	**LEV**	**CPM**	**CAZ**	**PTZ**	**ATM**			
HP22	R	R	R	R	R	R	R	R	R	R	R	S	**+**	**+**	XDR
HP25	S	R	R	R	R	S	I	R	R	S	S	I	**+**	**+**	MDR
HP49	R	R	R	R	R	R	R	R	R	S	R	R	**+**	**+**	XDR
HP61	I	R	R	R	R	R	I	R	R	I	S	I	**+**	**+**	MDR
HP64	R	R	R	R	R	S	I	R	I	S	S	R	**+**	−	MDR
HP77	R	R	R	R	R	R	R	R	R	R	R	I	**+**	**+**	XDR
HP78	R	R	S	R	R	R	R	R	R	I	R	S	**+**	**+**	XDR
HP81	R	R	R	R	R	R	R	R	R	R	R	R	**+**	**+**	XDR

Abbreviations: AK, amikacin; ATM, aztreonam; CAZ, ceftazidime; CIP, ciprofloxacin; CPM, cefepime; DOR, doripenem; I, intermediate; IMI, imipenem; LEV, levofloxacin; MEM, meropenem; PRL, piperacillin; PTZ, piperacillin–tazobactam; R, resistant; S, susceptible; TN, tobramycin.

**Table 5:** Summary of the prevalence of colistin heteroresistance in *P. aeruginosa* in other study.

	**Number of samples/HR**	**Prevalence of heteroresistance (%)**	**Frequency**	**Stability**	**Refs**
*P. aeruginosa*	231/9	4%	3.61 × 10^−8^ to 7.06 × 10^−6^	Stable	[6]
	143/29	26%	≥1 × 10^−7^	—	[14]
	152/41	27%	1.8 × 10^−7^ to 3 × 10^−4^	—	[26]
	24/1	4.2%	2.1 × 10^−7^ to 2.6 × 10^−4^	Stable	[27]
	66/8	12.9%	3 × 10^−6^ to 6.1 × 10^−3^	Stable	Our study

## Data Availability

The data that support the findings of this study are available from the corresponding author upon reasonable request.
