# Supplementary material for: Detection of Colistin Heteroresistance in Carbapenem-Resistant Pseudomonas aeruginosa Clinical Isolates in Iran
Source: Int J Microbiol. 2025 Oct 6;2025:5571153. doi: 10.1155/ijm/5571153 (PMC12517994; doi:10.1155/ijm/5571153)
Supplement: Supporting Information — Additional supporting information can be found online in the Supporting Information section. The MIC values of 66 CRPA isolates are briefly mentioned in Table S1. [file 5571153.f1.docx]

Supplementary table 1: The MIC values of 66 CRPA isolates

| **MIC *n (%)***  **Isolates** | | | | | | |
| --- | --- | --- | --- | --- | --- | --- |
| **CIP MEM IMI CAZ CO** | | | | | | |
| P1 | 32 | 16 | 4 | 4 | 1 |  |
| P3 | 32 | 128 | 64 | 32 | 0.5 |  |
| P5 | 32 | 8 | 8 | 4 | 0.5 |  |
| P6 | 0.5 | 16 | 4 | 4 | 1 |  |
| P7 | 32 | 8 | 16 | 8 | 1 |  |
| P8 | 64 | 8 | 16 | 8 | 1 |  |
| P10 | 0.5 | 8 | 32 | 8 | 0.5 |  |
| P18 | 32 | 32 | 128 | 64 | 0.5 |  |
| P20 | 32 | 8 | 8 | 128 | 1 |  |
| P21 | 32 | 8 | 8 | 64 | 0.5 |  |
| P22 | 64 | 256 | 64 | 64 | 1 |  |
| P23 | 64 | 8 | 16 | 128 | 0.5 |  |
| P25 | 64 | 64 | 16 | 8 | 0.5 |  |
| P26 | 128 | 4 | 16 | 32 | 0.5 |  |
| P27 | 8 | 8 | 16 | 256 | 1 |  |
| P28 | 16 | 16 | 32 | 32 | 1 |  |
| P29 | 16 | 8 | 16 | 4 | 0.5 |  |
| P30 | 256 | 32 | 32 | 32 | 1 |  |
| P31 | 32 | 8 | 16 | 64 | 1 |  |
| P32 | 0.25 | 8 | 2 | 32 | 1 |  |
| P33 | 128 | 16 | 8 | 128 | 1 |  |
| P34 | 128 | 64 | 32 | 128 | 0.5 |  |
| P35 | 16 | 8 | 0.5 | 2 | 1 |  |
| P36 | 8 | 8 | 4 | 64 | 0.5 |  |
| P37 | 64 | 16 | 8 | 64 | 1 |  |
| P38 | 8 | 8 | 4 | 128 | 1 |  |
| P39 | 16 | 16 | 4 | 64 | 0.5 |  |
| P40 | 128 | 128 | 64 | 128 | 0.5 |  |
| P41 | 128 | 64 | 64 | 32 | 1 |  |
| P43 | 8 | 8 | 256 | 32 | 128 |  |
| P45 | 0.5 | 4 | 8 | 4 | 1 |  |
| P47 | 32 | 8 | 16 | 4 | 4 |  |
| P49 | 64 | 4 | 16 | 2 | 0.5 |  |
| P50 | 32 | 4 | 16 | 2 | 0.5 |  |
| P51 | 64 | 4 | 4 | 8 | 1 |  |
| P53 | 64 | 16 | 8 | 8 | 1 |  |
| P54 | 64 | 8 | 8 | 8 | 1 |  |
| P55 | 64 | 16 | 8 | 8 | 1 |  |
| P56 | 64 | 8 | 4 | 8 | 4 |  |
| P57 | 0.5 | 4 | 8 | 64 | 2 |  |
| P58 | 0.25 | 8 | 4 | 32 | 16 |  |
| P59 | 0.25 | 8 | 8 | 128 | 2 |  |
| P61 | 16 | 16 | 32 | 8 | 1 |  |
| P64 | 32 | 8 | 16 | 4 | 0.5 |  |
| P65 | 16 | 16 | 16 | 128 | 0.5 |  |
| P68 | 8 | 256 | 256 | 256 | 0.5 |  |
| P69 | 32 | 32 | 16 | 256 | 0.5 |  |
| P70 | 0.5 | 256 | 128 | 32 | 0.5 |  |
| P74 | 64 | 16 | 16 | 128 | 0.5 |  |
| P75 | 16 | 16 | 8 | 256 | 0.5 |  |
| P76 | 32 | 128 | 128 | 256 | 0.5 |  |
| P77 | 32 | 256 | 256 | 256 | 0.5 |  |
| P78 | 64 | 64 | 2 | 8 | 0.5 |  |
| P79 | 32 | 128 | 32 | 256 | 0.5 |  |
| P81 | 64 | 256 | 256 | 256 | 1 |  |
| P82 | 64 | 256 | 256 | 256 | 0.5 |  |
| P85 | 0.5 | 32 | 256 | 2 | 0.5 |  |
| P86 | 8 | 128 | 256 | 256 | 0.5 |  |
| P87 | 0.25 | 128 | 128 | 32 | 0.5 |  |
| P88 | 0.25 | 16 | 16 | 2 | 0.5 |  |
| P89 | 0.5 | 128 | 256 | 128 | 0.5 |  |
| P91 | 256 | 128 | 64 | 128 | 0.5 |  |
| P92 | 0.5 | 32 | 8 | 256 | 0.5 |  |
| P98 | 0.5 | 256 | 256 | 64 | 0.5 |  |
